# Metachronous cholangiocarcinoma following pancreaticoduodenectomy and the potential role of a migrated pancreaticojejunal stent: a case report and review of the literature

**DOI:** 10.3389/fonc.2026.1795555

**Published:** 2026-06-22

**Authors:** Jing Chen, Feng Yi Wang, Yi Xin Huang, Li Ye Zhu

**Affiliations:** The Second Affiliated Hospital of Jiaxing University, Jiaxing, China

**Keywords:** case report, cholangiocarcinoma, cholangitis, pancreaticojejunostomy stent, pancreatoduodenectomy

## Abstract

Metachronous cholangiocarcinoma following curative surgery for periampullary neoplasia is a rare but serious long-term complication that poses significant diagnostic and management challenges. We report the case of a 62-year-old male who developed hilar cholangiocarcinoma 9 years after undergoing a pancreaticoduodenectomy (Whipple procedure) for an ampullary adenoma. The patient was initially diagnosed with a duodenal papillary villous tubular adenoma showing moderate dysplasia in 2015 and underwent curative resection, with no evidence of recurrence during subsequent follow-up. In 2024, he presented with obstructive jaundice and imaging findings revealing a hepatic hilar mass with intrahepatic biliary dilation. A biopsy confirmed a poorly differentiated cholangiocarcinoma (Klatskin tumor). Crucially, intraoperative exploration identified migration of the internal pancreaticojejunostomy stent to a site adjacent to the biliary-enteric anastomosis. Immunohistochemical analysis demonstrated wild-type P53 expression and proficient mismatch repair function, supporting a sporadic rather than hereditary origin. This case underscores the importance of considering second primary malignancies in patients with late-onset biliary obstruction after pancreaticoduodenectomy. We propose that the migrated and retained pancreatic stent may have acted as a chronic local irritant, potentially exacerbating inflammation and contributing to carcinogenesis within the biliary-enteric anastomotic milieu, thereby highlighting a possible long-term risk associated with indwelling surgical stents.

## Introduction

The pancreaticoduodenectomy (Whipple procedure) is the standard curative treatment for periampullary neoplasms. While long-term follow-up focuses on recurrence and surgical complications like pancreatic insufficiency or anastomotic stricture, the development of a metachronous second primary malignancy in the biliary tract is exceedingly rare. Cholangiocarcinoma is known to arise in settings of chronic biliary inflammation (1). Biliary-enteric anastomosis has been implicated as a potential risk factor for late-onset cholangiocarcinoma, with reported intervals often exceeding a decade (2). This report presents a case of hilar cholangiocarcinoma occurring just nine years post-Whipple, in which a migrated pancreatic stent was identified as a possible contributing factor in the carcinogenic process, underscoring an under-recognized long-term complication.

## Case description

A 62-year-old male with no significant past medical or family history of cancer was admitted in April 2015 with upper abdominal pain and jaundice. Laboratory tests revealed obstructive jaundice (Total Bilirubin: 199.7 μmol/L, Direct Bilirubin: 163.2 μmol/L) and signs of infection (WBC: 14.14×10^9^/L, hs-CRP: 63.79 mg/L). Baseline CA19–9 was 15.3 U/mL (institutional upper limit of normal: 37 U/mL). Abdominal CT demonstrated dilation of the common bile duct and intrahepatic bile ducts, indicating biliary obstruction. The following day, he developed acute cholangitis with chills and high fever (39.0 °C), prompting emergency Endoscopic Retrograde Cholangiopancreatography (ERCP). ERCP revealed a swollen duodenal papilla; biopsies were taken and a biliary stent was placed. A follow-up contrast-enhanced CT confirmed the stent was *in situ* with persistent biliary dilation. Histopathology of the ERCP biopsy showed moderate to severe dysplasia (**Figure 1**). After excluding surgical contraindications, the patient underwent a Whipple procedure on June 20, 2015. An internal pancreaticojejunostomy stent was placed during the surgery. The stent was a 5 Fr, 8-cm plastic stent made of polyethylene (manufacturer and product name not documented in the medical record). According to our institutional protocol, pancreatic stents are generally left in place without scheduled removal, as routine endoscopic retrieval is not standard and spontaneous migration is common. Final pathology confirmed a duodenal papillary villous tubular adenoma with moderate dysplasia (**Figure 1**). The patient’s postoperative recovery was satisfactory.

**Figure 1:**
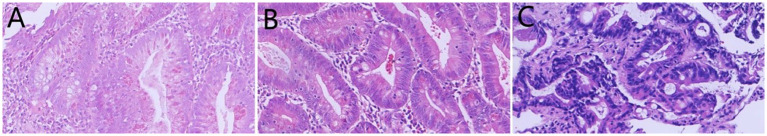
Histopathological findings. **(A)** ERCP biopsy: moderate to severe dysplasia, with nuclear hyperchromasia, increased nuclear-to-cytoplasmic ratio, and loss of polarity (H&E, ×400). **(B)** Whipple specimen: villous tubular adenoma of the duodenal papilla with moderate dysplasia (H&E, ×400). **(C)** Hilar mass biopsy: poorly differentiated adenocarcinoma, with irregular glands, nuclear pleomorphism, and frequent mitoses (H&E, ×400).

Over the nine-year follow-up period following the Whipple procedure, the patient experienced a total of 8 documented episodes of acute cholangitis (including the immediate postoperative episode). These episodes were diagnosed based on the presence of fever (>38.5 °C), right upper quadrant abdominal pain, and elevated liver enzymes (ALT and AST >2× upper limit of normal), consistent with Tokyo Guidelines criteria. All episodes were treated conservatively with intravenous antibiotics (piperacillin-tazobactam or ceftriaxone); no endoscopic or percutaneous drainage was required. Cross-sectional imaging (contrast-enhanced CT) was performed during 3 of these 8 episodes (at years 2, 4, and 7 post-surgery), each time demonstrating persistent partial migration of the pancreatic stent at the biliary-enteric anastomosis without evidence of anastomotic stricture or biliary stones. A follow-up abdominal CT performed two years after the initial surgery (in 2017) showed partial migration of the pancreatic stent to the level of the biliary-enteric anastomosis, this was the first documented imaging finding of stent migration. No dedicated imaging was performed during the remaining 5 episodes (**Table 1**).

**Table 1:** Timeline of key clinical events over the 9-year follow-up period.

Time point	Event	Clinical/laboratory findings	Imaging	Intervention
April 2015 (baseline)	Initial presentation	Obstructive jaundice, CA19-9 15.3 U/mL; ERCP biopsy: moderate-severe dysplasia	CT: dilated bile ducts	Biliary stent placement
June 2015	Whipple procedure	Duodenal papillary villous tubular adenoma with moderate dysplasia	None	Pancreaticojejunostomy with 5Fr, 8cm polyethylene stent placed
Jan 2016 (postop 9 mo)	Routine follow-up	Asymptomatic	CT: normal bile duct, no visible stent	None
2017 (postop 2 yr)	Cholangitis episode (1st documented migration)	Fever, elevated LFTs	CT: partial stent migration to biliary-enteric anastomosis	Antibiotics alone
Years 2–9	Additional 7 cholangitis episodes (total 8)	Fever, elevated LFTs; all resolved with antibiotics	CT performed in 3 of 8 episodes: persistent stent migration, no anastomotic stricture	Antibiotics; no drainage
Oct 2024 (postop 9 yr)	Cancer diagnosis	Jaundice, CA19-9 41.11 U/mL; biopsy: poorly differentiated adenocarcinoma	CT/MRI: hilar mass (Klatskin tumor)	PTCD, then surgical exploration
Oct 2024	Surgery	Stent found adjacent to biliary-enteric anastomosis	Cholangiogram: filling defect at hilum	Biopsy and T-tube drainage

Nine years after the initial surgery (in October 6, 2024), the patient was re-admitted with a 20-day history of jaundice and intermittent fever. Laboratory tests again indicated severe obstructive jaundice (Total Bilirubin: 296.1 μmol/L, Direct Bilirubin: 154.4 μmol/L) and active inflammation (WBC: 10.23×10^9^/L, hs-CRP: 60.94 mg/L). The tumor marker CA19–9 was elevated at 41.11 U/mL (upper limit of normal: 37 U/mL). Contrast-enhanced abdominal CT revealed significant wall thickening, enhancement, and a soft tissue nodule at the hepatic hilum, highly suspicious for cholangiocarcinoma, accompanied by intrahepatic biliary dilation (**Figure 2**). Subsequent abdominal MRI confirmed a hilar soft tissue nodule consistent with imaging features of hilar cholangiocarcinoma (Klatskin tumor). Percutaneous Transhepatic Biliary Drainage (PTCD) was performed to relieve the obstruction. Two weeks later, surgical exploration was performed. The tumor was found to invade the confluence of the right and left hepatic ducts (**Figure 3**). A critical finding was that the internal stent originally placed in the pancreaticojejunal anastomosis had migrated, with one end situated adjacent to the biliary-enteric anastomosis. A biopsy of the hilar tumor was taken, and T-tube drainage was established. Postoperative pathology confirmed a poorly differentiated adenocarcinoma of the bile duct (**Figure 1**). Immunohistochemistry showed wild-type P53 expression and positive staining for all mismatch repair proteins (MSH2, MSH6, MLH1, PMS2), indicating proficient mismatch repair function. Histological re-examination of the perianastomotic bile duct tissue adjacent to the stent revealed moderate to severe chronic inflammation with lymphocytic infiltration; no definitive foreign-body giant cells or granulomas were identified. Due to limited residual archival tissue after routine diagnostic processing, additional immunohistochemical evaluation (e.g., Ki-67, COX-2, IL-6) could not be performed. No adjuvant or neoadjuvant chemotherapy or radiotherapy was administered at any time point. The patient’s management consisted solely of surgical intervention (Whipple procedure in 2015 and subsequent exploratory surgery with biopsy and T−tube drainage in 2024) plus antibiotic therapy for episodes of cholangitis.

**Figure 2:**
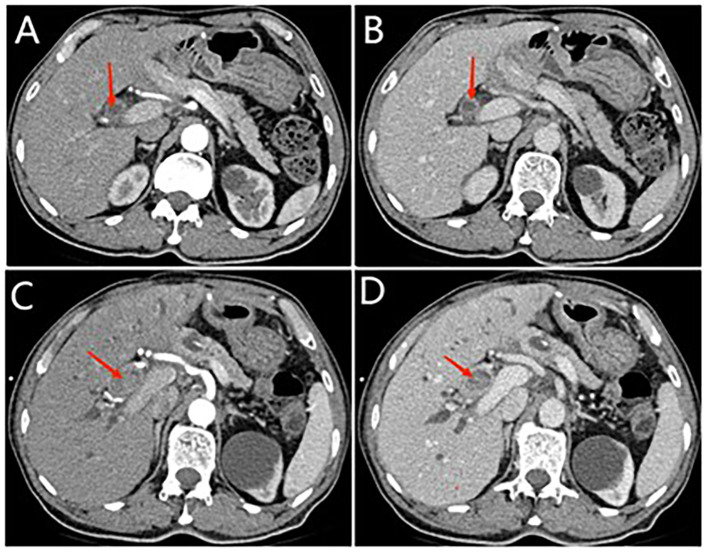
Serial abdominal CT changes at the hepatic hilum over 9 years. **(A, B)** January 2016: normal bile duct in arterial **(A)** and venous **(B)** phases; the migrated stent is not visible. **(C, D)** October 2024: soft tissue nodule at the hepatic hilum (red arrows) with heterogeneous enhancement and intrahepatic biliary dilation; no visible stent.

**Figure 3:**
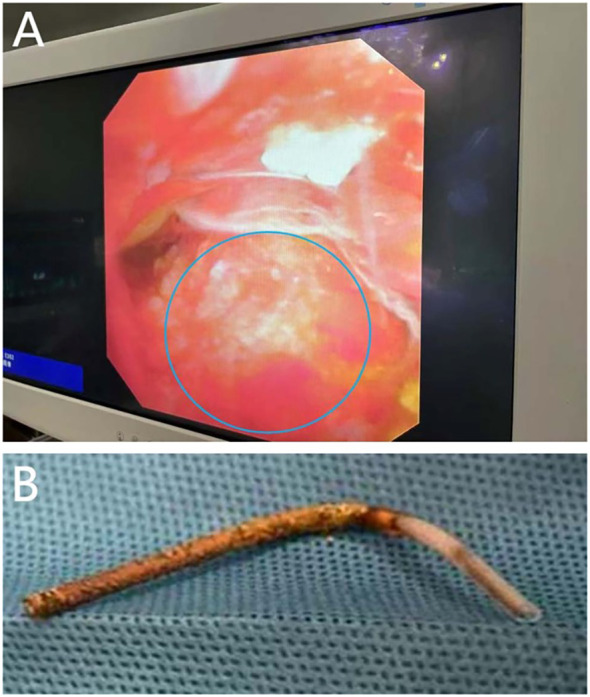
Intraoperative and cholangiographic findings. **(A)** Cholangiogram: irregular filling defect and narrowing at the confluence of the right and left hepatic ducts (arrow), consistent with hilar cholangiocarcinoma (Klatskin tumor); intrahepatic bile ducts are moderately dilated. **(B)** Retrieved pancreatic stent: 5 Fr, 8-cm straight plastic stent, originally placed at the pancreaticojejunostomy, found adjacent to the biliary-enteric anastomosis.

## Discussion

The central question posed by this case is why a second primary cholangiocarcinoma developed at the biliary-enteric anastomosis only nine years after a curative Whipple procedure. This rare event necessitates a systematic analysis beyond conventional recurrence, integrating the classic cholangiocarcinoma etiology, the known risks of biliary-enteric anastomosis, the specific features of this case, and the phenomenon of pancreatic stent migration.

The etiology of cholangiocarcinoma is closely linked to chronic biliary inflammation from various causes, such as primary sclerosing cholangitis, liver fluke infection, and choledochal cysts. The common pathophysiological core is a vicious cycle of “chronic inflammation-damage-repair” (3). A prolonged inflammatory microenvironment promotes biliary epithelial proliferation and, under the influence of factors like reactive oxygen species, drives the accumulation of genetic mutations leading to cancer (1). Notably, biliary-enteric anastomosis itself is considered a potential risk factor for inducing such chronic inflammation and subsequent late-onset cholangiocarcinoma (2). Literature documents this risk since Shields’ first report in 1977 of adenocarcinoma at a choledochoenteric anastomosis 14 years post-pancreatoduodenectomy (4). Subsequent case reports, with intervals ranging from 3 to 47 years (5–17) (**Supplementary Table 1**), and larger retrospective studies have substantiated this finding. For instance, a study by Hakamada et al. identified 8 cases of primary bile duct cancer among 108 patients (7.4%) within 1 to 20 years following sphincteroplasty (18). More definitively, a larger retrospective analysis by Tocchi et al. reported 55 cases of primary bile duct cancer among 1,003 patients (5.5%) at intervals of 132 to 218 months (approximately 11 to 18 years) after biliary-enteric anastomosis (2). These findings collectively suggest that biliary-enteric drainage, while therapeutic, may create a chronically inflamed milieu conducive to carcinogenesis.

Focusing on our patient, we have ruled out common hereditary predispositions. The immunohistochemical profile, wild-type P53 and proficient mismatch repair (pMMR), argues against hereditary syndromes like Lynch syndrome, supporting a sporadic origin (19, 20). This finding underscores the likelihood that the driver for carcinogenesis in this case was a postoperative, localized environmental alteration, aligning with the established long-term risk profile of biliary-enteric anastomosis.

However, the interval from initial surgery to cholangiocarcinoma diagnosis in our patient was 9 years, notably shorter than the average interval of approximately 13 years suggested by the literature (**Table 2**). Although this difference is not statistically testable in a single case, and the published intervals range widely from 3 to 47 years, the relatively short interval in our patient raises the possibility of an additional local accelerating factor. This temporal discrepancy is significant. Having excluded genetic accelerants, we must identify a local factor capable of significantly hastening the “chronic inflammation-carcinogenesis” sequence. This logically directs attention to the most distinctive finding in this case.

**Table 2:** Cholangiocarcinoma: clinical characteristics of patients from literature and present case.

Characteristic	Large-scale retrospective studies		Case reports (4–17)	Our case report
	Hakamada (18) (n=8)	Tocchi (2) (n=55)	(n=21)	(n=1)
Age (yr)	59.9 ± 11.8	55.0 ± 10.5	61.4 ± 9.2	70
Gender				
Male	6	17	4	1
Female	2	38	10	0
Surgical procedure				
Transduodenal sphincteroplasty	8	36	0	0
Choledochoduodenostomy	0	16	14	0
Choledochojejunostomy	0	0	5	0
Hepaticojejunostomy	0	3	1	0
Pancreatoduodenectomy	0	0	1	1
Follow-up (months)	133.5 ± 73.5	111.2 ± 31.3	291.8 ± 125.3	108
Cholangitis, n	7	40	9	1

The gender data for the case report cohort is incomplete due to unreported information in some original studies.

It is noteworthy that migration of the internal pancreaticojejunal (PJ) stent into the biliary tract is a recognized, though often overlooked, long-term complication. A radiographic review of 802 patients found a 16.8% incidence of PJ stent migration into the bile ducts. Among these 135 patients, 40 (29.6%) developed stent-induced complications including bile duct stricture, stone formation, and liver abscess (21). That study identified longer stent retention time in the bile ducts as a risk factor for such complications. This evidence confirms that stent migration and retention is not rare and is sufficient to induce local structural changes and inflammation. Importantly, existing literature has hypothesized that pancreatic stent migration into the bile duct, by causing chronic cholangitis, bile duct stricture, and stone formation, may potentially predispose to cholangiocarcinoma. However, to the best of our knowledge, no prior case report has directly implicated a migrated surgical stent as a confirmed causative or contributing factor for cholangiocarcinoma at a biliary-enteric anastomosis.

The core pathological process instigated by a migrated stent is chronic recurrent cholangitis. Substantial evidence confirms that persistent biliary inflammation is central to cholangiocarcinoma development. The stent-related complications mentioned above are direct manifestations or consequences of chronic cholangitis. In our patient, the recurrent episodes of cholangitis over the nine-year period could have resulted from either the migrated stent itself or from an independent anastomotic stricture. We cannot completely exclude the possibility that an anastomotic stricture, in the absence of the stent, caused the chronic cholangitis. However, the documented stent migration detected on CT two years postoperatively, together with the intraoperative finding of the stent adjacent to the anastomosis, suggests that the stent was a likely contributor to the persistent inflammatory state.

The absence of classic foreign-body giant cells or granulomas in the perianastomotic tissue warrants further mechanistic consideration. Chronic biliary inflammation leading to cholangiocarcinoma is well recognized in conditions such as primary sclerosing cholangitis (PSC), hepatolithiasis, and parasitic cholangitis (e.g., Clonorchis sinensis), none of which typically produce a granulomatous response (22, 23). Instead, these conditions promote carcinogenesis through persistent non-granulomatous mechanisms including mechanical irritation, bile stasis, recurrent bacterial cholangitis, and accumulation of toxic bile acids. In our patient, the migrated stent likely contributed through several such pathways: (1) chronic low-grade mechanical trauma to the biliary epithelium at the anastomotic site; (2) intermittent bile flow obstruction leading to localized stasis; (3) serving as a nidus for bacterial biofilm formation, perpetuating recurrent cholangitis; and (4) secondary bile acid-mediated epithelial injury. These mechanisms, rather than a florid foreign-body reaction, are consistent with the absence of granulomas and align with established models of inflammation-associated cholangiocarcinogenesis. Thus, the lack of giant cells does not undermine the hypothesis but instead suggests a more indolent, low-grade inflammatory driver, one that is clinically reflected by the patient’s multiple cholangitis episodes over nine years.

We acknowledge that additional immunohistochemical markers (e.g., Ki-67 to assess epithelial proliferation, COX-2 or IL-6 as inflammatory mediators) could provide further support for the proposed mechanism. However, due to limited residual tissue after routine diagnostic processing, such analyses were not feasible in this case. Future studies encountering similar scenarios are encouraged to perform such ancillary investigations.

Therefore, while the theoretical risk has been acknowledged, this case appears to be the first reported instance in which a migrated pancreaticojejunal stent is clinically observed and proposed as a potential accelerant of carcinogenesis in the biliary-enteric anastomotic setting. The migrated pancreatic stent found in our patient, having remained in a critical biliary location for 7 years (from 2017 to 2024), may represent a contributing factor that links the established general risk associated with biliary-enteric anastomosis and the specific, accelerated carcinogenesis observed in this case. This prolonged retention aligns perfectly with the recognized high-risk profile for such complications. We therefore propose the following plausible pathogenic sequence: the migration and long-term retention of the pancreatic stent within the biliary tract could act as an additive local irritant, potentially exacerbating chronic recurrent cholangitis and structural biliary changes, building upon the baseline inflammatory environment created by the biliary-enteric anastomosis. Within this sustained and intensified inflammatory and injurious microenvironment over nine years, the stent might drives the malignant transformation of the biliary epithelium, culminating in the development of hilar cholangiocarcinoma. This proposed mechanism coherently aligns with the recognized pattern of delayed carcinogenesis following biliary-enteric anastomosis while simultaneously accounting for the precisely localized site of the cancer and the relatively abbreviated timeline evident in this patient.

This case report has several inherent limitations. Primarily, the nature of a single case report precludes definitive causal inference. While we propose a compelling pathogenic sequence linking the migrated pancreatic stent to accelerated carcinogenesis, this association remains observational and hypothetical. Second, our retrospective analysis relies on historical clinical data, and detailed longitudinal imaging to precisely document the timing and consequences of stent migration prior to cancer diagnosis was not available. Specifically, although a CT scan performed two years postoperatively confirmed stent migration, no dedicated cross-sectional imaging was performed during most episodes of cholangitis in the intervening years; therefore, we cannot determine whether the stent remained stable or migrated further. Third, although we ruled out common hereditary syndromes via immunohistochemistry, we cannot entirely exclude the potential contribution of other undetected genetic or environmental co-factors in this individual patient. The impact of the patient’s recurrent episodes of cholangitis, while highlighted, is also difficult to quantify precisely in relation to the stent’s role. Fourth, the histopathological examination of the perianastomotic bile duct revealed chronic lymphocytic inflammation but no definitive foreign-body giant cells; the absence of such findings does not preclude a chronic irritant effect, but it underscores the need for further mechanistic studies.

Future directions should focus on validating and refining this proposed mechanism. Prospective, long-term registries for patients undergoing pancreaticoduodenectomy with indwelling stents are needed to establish the true incidence and risk factors for stent migration and subsequent complications. Multicenter collaborative studies could aggregate similar cases to strengthen the epidemiological link. Furthermore, basic science research utilizing *in vitro* or animal models could directly investigate the molecular and cellular consequences of chronic biliary epithelial exposure to foreign body materials, testing the hypothesis of inflammation amplification and accelerated transformation. Such efforts are crucial to determine whether a change in clinical practice, such as the routine use of biodegradable stents or protocolized imaging surveillance for stent migration, is warranted to mitigate this potential long-term oncological risk.

## Conclusion

This case report highlights a metachronous cholangiocarcinoma developing at an accelerated pace (9 years) following Whipple surgery, with a migrated pancreaticojejunal stent implicated as a potential contributing factor to the carcinogenic process. The findings suggest that while the biliary-enteric anastomosis establishes a baseline risk through chronic inflammation, a retained foreign body like a stent might amplify this process, possibly shortening the carcinogenesis window. This underscores the imperative for heightened long-term vigilance, including consideration of the fate of indwelling stents, in patients who have undergone pancreaticoduodenectomy. It also calls for a re-evaluation of the long-term safety of non-absorbable internal stents and promotes strategies like using biodegradable alternatives or implementing protocols for monitoring and potentially retrieving migrated stents.

## Data Availability

The original contributions presented in the study are included in the article/**Supplementary Material**. Further inquiries can be directed to the corresponding author.
