# Supplementary material for: Metachronous cholangiocarcinoma following pancreaticoduodenectomy and the potential role of a migrated pancreaticojejunal stent: a case report and review of the literature
Source: Front Oncol. 2026 Jun 22;16:1795555. doi: 10.3389/fonc.2026.1795555 (PMC13333422; doi:10.3389/fonc.2026.1795555)
Supplement: Supplementary file 1 [file Table1.docx]

Table 1 Characteristics of patients with a history of biliary surgery who develop late cholangiocarcinoma

| Year | Gender | Age（year） | Cause | Surgical method | interval（year） |
| --- | --- | --- | --- | --- | --- |
| 1977^[4]^ | M | 35 | Chronic pancreatitis | Pancreatoduodenectomy | 14 |
| 1983^[5]^ | F | 54 | CBD stenosis | Choledochojejunostomy | 9 |
| 1984^[6]^ | NC | NC | CBD stone | Choledochoduodenostomy | NC |
|  | NC | NC | CBD stone | Choledochoduodenostomy | NC |
| 1986^[7]^ | M | 64 | CBD transection | Choledochoenterostomy | 17 |
|  | F | 52 | Dysfunction of Oddi sphincter | Choledochoduodenostomy | 19 |
| 1994^[8]^ | NC | NC | Benign diseases of CBD | Cholecystojejunostomy | 11 |
|  | NC | NC | Benign diseases of CBD | Choledochoduodenostomy | 3 |
| 1997^[9]^ | F | 71 | Benign diseases of CBD | Choledochoduodenostomy | 38 |
| 1999^[10]^ | NC | 71 | Iatrogenic bile duct injury | Choledochojejunostomy | 40 |
|  | NC | 66 | Iatrogenic bile duct injury | Choledochojejunostomy | 25 |
|  | NC | 57 | Iatrogenic bile duct injury | Choledochoduodenostomy | 27 |
| 2002^[11]^ | F | 51 | Iatrogenic bile duct injury | Choledochoduodenostomy | 26 |
|  | F | 73 | Iatrogenic bile duct injury | Choledochoenterostomy | 39 |
|  | M | 72 | CBD stone | Choledochoduodenostomy | 15 |
| 2003^[12]^ | F | 67 | CBD stone | Choledochoduodenostomy | 21 |
| 2004^[13]^ | F | 68 | CBD stone | Choledochoduodenostomy | 12 |
| 2012^[14]^ | F | 62 | Congenital biliary dilatation | Choledochoduodenostomy | 47 |
| 2014^[15]^ | F | 67 | Congenital biliary dilatation | Choledochojejunostomy | 23 |
| 2016^[16]^ | F | 41 | Congenital biliary dilatation | Cyst-to-duct anastomosis Choledochoduodenostomy | 38 |
| 2019^[17]^ | M | 72 | CBD stone | Choledochoduodenostomy | 38 |
| 2024^*^ | M | 70 | Duodenal papillary adenoma | Pancreatoduodenectomy | 9 |

NC: unclear. CBD: Common bile duct. *: present case
